# Supplementary material for: The Role of the Pathogen Dose and PI3Kγ in Immunometabolic Reprogramming of Microglia for Innate Immune Memory
Source: Int J Mol Sci. 2021 Mar 4;22(5):2578. doi: 10.3390/ijms22052578 (PMC7961448; doi:10.3390/ijms22052578)
Supplement: Supplementary file 1 [file ijms-22-02578-s001.pdf]

Supplementary Figures

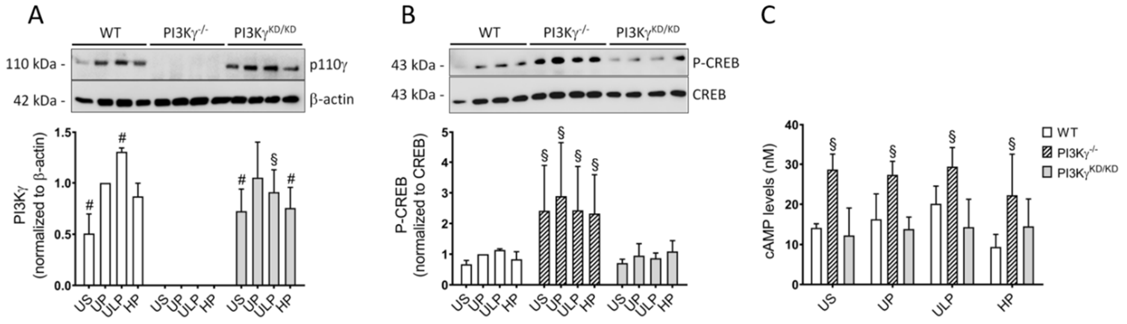

Supplementary Figure 1. Impact of microglia stimulation by LPS including dose-dependent priming of PI3Kγ expression and effects of lipid kinase dependent and independent PI3Kγ signaling on parameters of intracellular signaling. Primary microglial cells (wild-type, open columns; PI3Kγ$^{-/-}$, hatched columns; PI3Kγ$^{KD/KD}$, dark gray columns) were primed initially by ultra-low (ULP, 1 fg/mL) or high (HP, 100 ng/mL) doses of LPS, followed by a second stimulation (day 6) with 100 ng/mL LPS. Lysates were collected 24h after the second stimulus and analyzed for (A) p110γ protein expression (n=6) and (B) phospho-CREB protein expression (n=5) using Western blotting and quantified (wild-type unprimed cells assigned as 1.0). (C) cAMP levels (n=5) were assayed using the cAMP GloAssayKit. Data are shown as mean + SD, $^{\#\S}$ $p < 0.05$, $^{\#}$ vs. unprimed condition, § vs. wild-type strain. US, unstimulated; UP, unprimed.

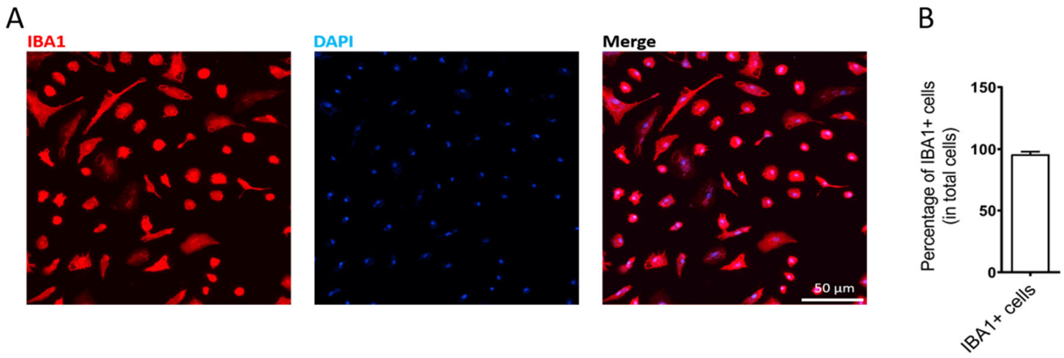

Supplementary Figure 2. Purity of primary microglial cell culture assessed by the percentage of IBA1-positive cells (A, red color) in total cells indicated by immunocytochemical staining using DAPI (blue color). N=6, at least 5 randomly selected fields were used for quantification (B). Data are shown as mean + SD.

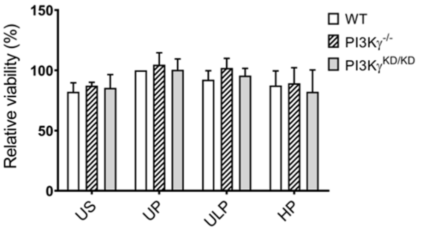

Supplementary Figure 3. Cell viability in primary microglial cell culture primed by LPS.

Primary microglial cells (wild-type, open columns; PI3K$\gamma^{-/-}$, hatched columns; PI3K$\gamma^{KD/KD}$, dark gray columns) were primed initially by ultra-low (ULP, 1 fg/mL) or high (HP, 100 ng/ml) doses of LPS, followed by a second stimulation (day 6) with 100 ng/ml LPS. Cell viability was measured using MTT assay (n=4, wild-type cells assigned as 100%). Data are shown as mean + SD, $^{\#\,\S}$ $p < 0.05$, $^{\#}$ vs. unprimed condition of wild-type microglia, § vs. wild-type strain. US, unstimulated; UP, unprimed.
